# Incidence and Determinants of Acute Mountain Sickness in Mount Kinabalu, Malaysia

**DOI:** 10.1089/ham.2020.0026

**Published:** 2020-09-08

**Authors:** Su Lan Yang, Nor At'fina Ibrahim, Grazele Jenarun, Houng Bang Liew

**Affiliations:** ^1^ Centre for Clinical Epidemiology, Institute for Clinical Research, National Institutes of Health Malaysia, Shah Alam, Malaysia.; ^2^ Emergency & Trauma Department, Hospital Temerloh, Temerloh, Malaysia.; ^3^ Clinical Research Centre, Hospital Queen Elizabeth II, Kota Kinabalu, Malaysia.

**Keywords:** acute mountain sickness, high-altitude sickness, incidence, Malaysia, Mount Kinabalu

## Abstract

Yang, Su Lan, Nor At'fina Ibrahim, Grazele Jenarun, and Houng Bang Liew. Incidence and determinants of acute mountain sickness in Mount Kinabalu, Malaysia. *High Alt Med Biol*. 21:265–272, 2020.

***Background:*** Acute mountain sickness (AMS) is the most common type of high-altitude sickness. The incidence of AMS varies by mountain location, trail characteristics, and study design. The lack of local epidemiology data has driven us to investigate the incidence and severity of AMS and its associated factors at Mount Kinabalu, Malaysia.

***Methods:*** A cohort study was conducted to collect data from climbers after days 1 (3272 m) and 2 (4095 m) of ascent. A self-administered questionnaire was used to explore climbers' demographic and climb characteristics, history of AMS, alcohol exposure, and AMS prevention measures. The Lake Louis score 2018 was used to assess the presence and severity of AMS (cutoff ≥3). Univariate and multivariable logistic regressions were performed to determine the factors associated with the development of AMS on day 2.

***Results:*** Data from 345 climbers were analyzed. The incidence of AMS was 23.9% (95% confidence interval [CI] 19.5%–28.7%) and 21.7% (95% CI 17.5%–26.3%) on days 1 and 2, respectively. The majority were mild cases. Experiencing AMS on day 1 (odds ratio [OR] = 12.88; 95% CI 6.71–24.75), alcohol consumption (OR = 3.73; 95% CI 1.66–8.39), receiving guide advice on day 1 (OR = 0.49; 95% CI 0.26–0.93), and age (OR = 0.96; 95% CI 0.93–0.99) were significant determinants of AMS at Mount Kinabalu. Gender, history of AMS, past exposure to high altitude, ascending time, water intake, acetazolamide use, physical fitness, pulse rate, and peripheral capillary oxygen saturation (SpO_2_) were not associated with AMS at Mount Kinabalu.

***Conclusion:*** Future analysis with age strata is required to ascertain the association of age with AMS. Our research has signposted a strong call for collaborative efforts to improve the provision of hiking advice and discourage alcohol sales to mitigate the risk of AMS among Mount Kinabalu climbers.

## Introduction

Acute mountain sickness (AMS) is the most common type of high-altitude sicknesses. AMS is an unpleasant and painful experience for climbers, with a possibility to develop into life-threatening high-altitude cerebral edema (HACE) (Luks et al., 2010; Bärtsch and Swenson, 2013). Symptoms such as headache, lethargy, dizziness, and nausea and vomiting occur within 6–12 hours after exposure to an altitude >2500 m (Roach et al., 1993, 2018). The incidence of AMS ranges between 28% and 50% (Mairer et al., 2010; Wang et al., 2010; McDevitt et al., 2014; Chan et al., 2016; Gonggalanzi et al., 2016; Horiuchi et al., 2016) globally, and the variation is due to differences in study design, mode of ascent, geographical location of the mountain, and sleeping altitude (Mairer et al., 2009; Waeber et al., 2015).

At 4095 m, Mount Kinabalu is the highest scalable mountain in Malaysia and South East Asia. Mount Kinabalu has unique mountaineering characteristics, including a diverse climate, distinctive terrain, and rapid ascent to the summit, making the hike to the summit one of the most popular climbing routes in the region. Every year, >29,000 climbers flock to conquer Mount Kinabalu—exposing themselves to the risk of high-altitude sicknesses. The 2-day hike starts from a tropical rainforest climate at Timpohon Gate (1866 m) and ascends rapidly to a sleeping altitude at Laban Rata basecamp (3272 m) in 5–7 hours. This is followed by an ascent to the Low's Peak summit (4095 m) at the wee hours of the next day. The climb can be perilous as climbers need to achieve a drastic elevation gain to >3000 m within 24 hours, thus making them susceptible to AMS (Bärtsch and Swenson, 2013).

The incidence and risk factors of AMS have been extensively studied in mountains that offer multiday hikes. The hike schedule of Mount Kinabalu is akin to a 2-day-stint in a multiday hike where climbers ascend to sleeping altitude, take an acclimatization day hike the next day to a higher altitude, and descend to the same sleeping altitude. However, compared with a multiday hike, a climbing route that involves a shorter hike to the summit (e.g., 2 days 1 night) often offers a limited choice for overnight altitude, little time for sleep and recovery, and is more popular among touristy novice climbers (Mairer et al., 2010; Horiuchi et al., 2016). To our knowledge, there is no literature on the epidemiology of AMS, and official reports on AMS-related injuries at Mount Kinabalu. Therefore, it is pertinent to generate local epidemiology data to guide national park managers in the dissemination of information and recommendation to promote AMS awareness among climbers. In this study, we aim to investigate the incidence and severity of AMS and its associated factors at Mount Kinabalu, Malaysia.

## Materials and Methods

### Participants

This study was conducted for 8 days in November 2018. Inclusion criteria were climbers >18 years old who hiked from either Timpohon Gate or Kota Belud Gate regardless of a successful summit climb. Exclusion criteria were climbers who did not hike to Laban Rata basecamp in 1 day. Climbers who met the criteria and consented to participate in this study were recruited.

There were two data collection points—days 1 and 2—for each participant at Laban Rata basecamp (3272 m). The first data collection took place when climbers completed their first-day ascent to Laban Rata; the second data collection took place on day 2 when climbers arrived back at Laban Rata after their descent from the summit. Resting pulse rate and peripheral capillary oxygen saturation (SpO_2_) were collected on both days. Climbers answered a self-administered questionnaire on day 1 only and the Lake Louise AMS questionnaire on both days. Participants were also prompted to inform researchers if any of their team members were unwell and unavailable for assessment at site. Efforts were taken to identify and recruit these unwell climbers to ensure representativeness.

### Materials

The self-administered questionnaire collected data on gender, nationality, age, knowledge, and concerns about AMS, history of AMS, exposure to high altitude in the past 2 months, alcohol consumption in the past 24 hours, fitness level, any chronic illnesses, total ascent time, water intake, and AMS preventive measures. All information was self-reported by study participants. Participants were also asked to recall at which pit stop did they start experiencing AMS symptoms, and if the mountain guides gave any AMS advice. We reported all core parameters in accordance with the STAR guideline for high-altitude clinical research (Maeder et al., 2018).

The Lake Louise AMS score (LLS) was used to summarize and grade the severity of AMS. LLS measured the severity of each AMS symptom—headache, gastrointestinal symptoms, fatigue, and dizziness or light-headedness—by grading it from 0 to 3 (0 for no symptom, 1 for mild, 2 for moderate, and 3 for severe and incapacitating symptoms) (Roach et al., 1993, 2018). AMS was defined as the presence of headache with at least a cumulative LLS of 3 (Roach et al., 2018). We further categorized the severity of AMS into mild (3–5 points), moderate (6–9 points), and severe AMS (10–12 points) (Roach et al., 2018).

### Statistical analysis

Continuous variables were presented using means with standard deviations, whereas categorical variables were presented using frequencies and percentages. The incidence of AMS was presented as a point estimate with 95% confidence intervals (CIs). Univariate and multivariable logistic regressions were performed to determine the factors associated with the development of AMS. Exposure variables with *p*-values <0.25 in univariate logistic regression were inputted into a multivariable logistic regression model through a stepwise forward method (Bursac et al., 2008; Ranganathan et al., 2017). Model fitness was assessed by the Hosmer–Lemeshow chi-square test where a *p*-value >0.05 indicates a good fit. Akaike's information criterion and Bayesian information criterion were presented for future model comparison where a smaller value indicates better model fitness. Odds ratios (ORs) were reported with 95% CI, and a 2-tailed *p*-value <0.05 was considered significant. All statistical analyses were performed using Stata version 15 (StataCorp LP).

## Results

A total of 356 climbers were recruited; data from 345 completed questionnaires were included in the logistic regression analysis. Table 1 summarizes the demographic characteristics of the study participants.

**Table 1.** Demographic Characteristics of All Climbers (*n* = 356)

Characteristics variable	n (%)	Mean* ± *SD
Age (years)^a^		35.2 ± 10.8
Gender male	222 (62.4)	
Nationality		
Malaysian	251 (70.5)	
Non-Malaysian	105 (29.5)	
Worry about AMS	187 (52.5)	
Past experience with AMS	48 (13.5)	
Ascend to >2500 m in the past 2 months	27 (7.6)	
Consumed alcohol in the past 24 hours	48 (13.5)	
Acetazolamide use	53 (14.9)	
Fitness level^b^		
Unfit	1 (0.3)	
Somewhat unfit	31 (8.7)	
Average	194 (54.4)	
Fit	116 (32.5)	
Very fit	14 (4.0)	
AMS knowledge^b^		
No	64 (18.0)	
Limited	165 (46.3)	
Sufficient	122 (34.3)	
Expert	5 (1.4)	
Chronic illnesses^c^		
Metabolic syndrome	19 (5.3)	
Bronchial asthma	18 (5.1)	
Cardiology illnesses	3 (0.8)	
Orthopedic illnesses	5 (1.4)	

^a^ Calculated based on 345 climbers, 11 missing values for age variable.

^b^ Fitness level and AMS knowledge were self-rated by climbers.

^c^ Metabolic syndrome included five diabetes mellitus, five dyslipidemia, eight hypertension, and one hyperuricemia. Cardiology illnesses included one acute coronary syndrome, one paroxysmal atrial tachycardia, and one arrhythmia. Orthopedic illnesses included two lumbar disk herniation, one femur platting, and two scoliosis.

AMS, acute mountain sickness; SD, standard deviation.

The incidence of AMS was 23.9% (95% CI 19.5%–28.7%) on day 1 and 21.7% (95% CI 17.5%–26.3%) on day 2 (Table 2). The incidence of AMS on both days was not significantly different. We found no case of HACE and high-altitude pulmonary edema throughout the study period. The majority of the AMS cases were mild, and few were of moderate intensity. The most common symptoms were fatigue and headache, followed by dizziness, and the least common were gastrointestinal symptoms.

**Table 2.** The Incidence of Acute Mountain Sickness and Acute Mountain Sickness Symptoms Reported by All Climbers (*n* = 356)

	Day 1		Day 2	
	n (%)	95% CI	n (%)	95% CI
AMS incidence (LLS ≥3)	85 (23.9)	19.5%–28.7%	77 (21.7)	17.5%–26.3%
Mild	71 (20.0)		59 (16.6)	
Moderate	14 (3.9)		17 (4.8)	
Severe	0 (0)		1 (0.3)	
Fatigue symptom^a^	275 (77.3)	72.5%–81.5%	253 (71.1)	66.0%–75.7%
Mild	164 (46.1)		146 (41.0)	
Moderate	101 (28.4)		96 (27.0)	
Incapacitating	10 (2.8)		11 (3.1)	
Headache symptom^a^	131 (36.7)	31.8%–42.0%	112 (31.5)	26.7%–36.6%
Mild	107 (30.1)		86 (24.2)	
Moderate	22 (6.2)		24 (6.7)	
Incapacitating	2 (0.5)		2 (0.6)	
Dizziness symptom^a^	106 (29.8)	25.1%–34.8%	90 (25.3)	20.8%–30.1%
Mild	92 (25.8)		77 (21.6)	
Moderate	14 (4.0)		13 (3.7)	
Incapacitating	0 (0)		0 (0)	
Gastrointestinal symptom^a^	45 (12.7)	9.4%–16.5%	51 (14.4)	10.8%–18.4%
Mild	33 (9.3)		39 (11.0)	
Moderate	6 (1.7)		7 (2.0)	
Incapacitating	6 (1.7)		5 (1.4)	

^a^ Symptoms reported by all climbers, not limited to AMS sufferers only.

CI, confidence interval; LLS, Lake Louise Score of AMS.

The mean pulse rate and SpO_2_ readings of climbers with and without AMS are presented in Table 3. We found a significant difference between the mean pulse rate (day 1) of climbers with and without AMS (105 ± 17 bpm vs 99 ± 14 bpm, *p* = 0.025). However, this difference dissipated in day 2. For SpO_2_, the mean readings differed significantly between climbers with and without AMS only on day 2 (88 ± 5% vs 89 ± 4%, *p* = 0.013).

**Table 3.** The Association Between Oximeter Variables and the Presence of Acute Mountain Sickness Among Climbers

Variables	With AMS		Without AMS		t Statistics (df)	p
	N	Mean* ± *SD	n	Mean* ± *SD		
Day 1 pulse rate (bpm)^a^	43	105 ± 17	154	99 ± 14	−2.2 (195)	0.025
Day 1 SpO_2_ (%)^a^	43	87 ± 5	154	87 ± 6	0.20 (195)	0.835
Day 2 pulse rate (bpm)	77	102 ± 14	278	102 ± 14	0.23 (353)	0.818
Day 2 SpO_2_ (%)	77	88 ± 5	278	89 ± 4	2.49 (353)	0.013

^a^ Day 1 pulse rate and SpO_2_ data calculated based on 197 climbers; day 2 data calculated based on 355 climbers.

SpO_2_, peripheral capillary oxygen saturation.

**FIG. 1.**
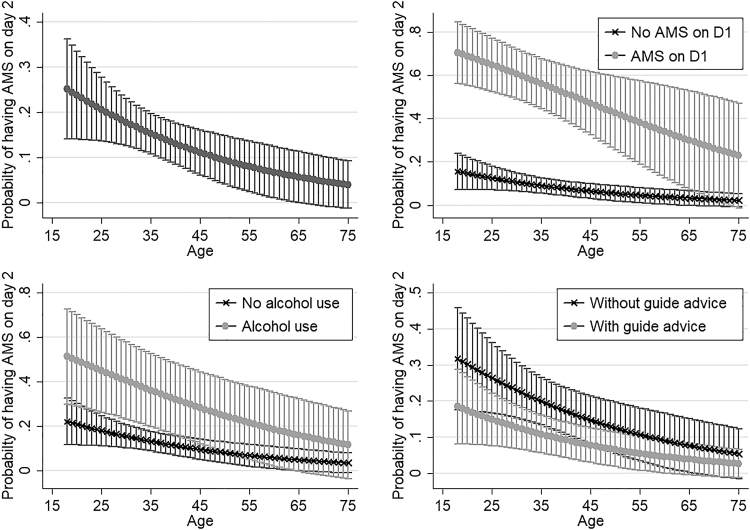
The effect of age, the presence of AMS on day 1, alcohol use and guide advice on the predicted probability (with 95% CI) of having AMS on day 2. AMS, acute mountain sickness; CI, confidence interval.

In the bivariate analysis, AMS on day 1, alcohol consumption in the past 24 hours, younger age, not receiving guide advice on day 1, and having insufficient knowledge about AMS were associated with AMS on day 2 (Table 4). In the multivariate analysis, climbers with AMS on day 1 (OR = 12.88; 95% CI 6.71–24.75) and having consumed alcohol in the past 24 hours (OR = 3.73; 95% CI 1.66–8.39) had higher odds of developing AMS on day 2. Climbers who received guide advice on day 1 (OR = 0.49; 95% CI 0.26–0.93) and those who were older (OR = 0.96; 95% CI 0.93–0.99) had lower odds of developing AMS on day 2. Figure 1 demonstrates the interaction between age and three other significant factors from the multivariable regression model (AMS on day 1, alcohol consumption, and received guide advice on day 1) on the probability of developing AMS on day 2.

**Table 4.** Univariate and Multivariable Logistic Regression of Factors Associated with Acute Mountain Sickness on Day 2

Predictors (*n* = 345)	Unadjusted OR (95% CI)	p	Adjusted OR (95% CI)	z	p
AMS on day 1	9.93 (5.61–17.60)	<0.001^a^	12.88 (6.71–24.75)	7.67	<0.001
Alcohol use	2.82 (1.48–5.38)	0.002^a^	3.73 (1.66–8.39)	3.18	0.001
Age	0.96 (0.93–0.98)	0.002^a^	0.96 (0.93,0.99)	−2.29	0.022
Guide advice on day 1	0.62 (0.37–1.04)	0.070^a^	0.49 (0.26–0.93)	−2.17	0.030
Guide advice on day 2	0.98 (0.58–1.67)	0.943	—		
Gender male	1.15 (0.68–1.95)	0.598	—		
History of AMS	0.58 (0.25–1.35)	0.207	—		
High-altitude exposure in the past 2 months	0.61 (0.20–1.82)	0.375	—		
Duration of ascent (minutes)^b^	1.00 (0.997–1.001)	0.535	—		
Fitness level^c^					
Unfit	(Ref.)		—		
Average	1.63 (0.59–4.48)	0.343	—		
Fit	1.48 (0.52–4.25)	0.462	—		
Very fit	0.9 (0.15–5.31)	0.907	—		
Water intake on day 1 (L)	1.06 (0.78–1.44)	0.709	—		
Water intake on day 2 (L)	1.35 (0.94–1.94)	0.097^a^	—		
Having sufficient-to-expert knowledge on AMS	0.52 (0.30,0.92)	0.024^a^	—		
Acetazolamide use	0.60 (0.27–1.34)	0.214^a^	—		

Model fit indicator: likelihood ratio test (df) = 89.16 (4), *p* < 0.001; the Hosmer–Lemeshow χ^2^ test (df) = 12.78 (8), *p* = 0.120; AIC = 276.92; BIC = 296.14.

^a^ Variables with *p*-value <0.25 in univariate analysis are selected for stepwise forward method in multivariable logistic regression.

^b^ Time taken from Timpohon gate (starting point) to Laban Rata Basecamp (overnight location) on day 1.

^c^ Somewhat unfit and unfit were recategorized into one group because there was only one participant who was “somewhat unfit.”

AIC, Akaike's information criterion; BIC, Bayesian information criterion; OR, odds ratio.

Water intake, AMS knowledge, AMS history, and acetazolamide use were included in the stepwise forward variable selection stage, but they were eventually removed from the final model as these variables did not achieve statistical significance in the multivariable analysis. The Hosmer–Lemeshow chi-square test (*p* = 0.12) showed good model fitness for this final regression model. Factors that were found not associated with AMS on day 2 were gender, high-altitude exposure in the past 2 months, duration of ascent, acetazolamide use, guide advice on day 2, and physical fitness.

## Discussion

### Incidence of AMS in Mount Kinabalu

The incidence of AMS in Mount Kinabalu was 23.9% (95% CI 19.5%–28.7%) on day 1 and 21.7% (95% CI 17.5%–26.3%) on day 2. The incidence of AMS at other locations of similar altitude and duration of the hike was 23% at Jiaming Lake, Taiwan (3350 m) (Hsu et al., 2015), 38% at the Eastern Alps (3454 m) (Mairer et al., 2009), 29% at Mount Fuji (3776 m) (Horiuchi et al., 2016), and 28%–36% at Jade Mountain (3952 m) (Kao et al., 2002; Wang et al., 2010). While the incidence at Mount Kinabalu was lower, significant differences cannot be established due to the lack of 95% CI reported in other studies. Although at lower altitudes, Mount Fuji and Jade Mountain recorded higher incidence of AMS. This may be due to the rapid ascent to sleeping altitude as both mountains have higher trailhead at 2305 and 2600 m, respectively. The higher incidence in the Eastern Alps study might be due to the higher sleeping altitude, smaller sample size, and possibly different climate (Mairer et al., 2009).

### Symptoms of AMS

Fatigue was the most prevalent AMS symptom with almost one-third of the climbers reported having moderate or incapacitating fatigue. Fatigue is a nonspecific symptom and can be a normal experience during strenuous exercise. As the cutoff value for the LLS is ≥3 points including headache, the fatigue score might lead to an overestimation of AMS incidence. Interestingly, among those who had AMS, 5.3% were unaware that they had AMS. This finding was not surprising as 64% of the climbers claimed to have zero to limited knowledge about AMS. Limited knowledge and awareness of AMS may have resulted in climbers' inability to recognize the symptoms, thereby exposing themselves to potential mountain injuries.

### Age may be protective against AMS

Previous literature showed conflicting and inconclusive associations between age and AMS (Wang et al., 2010; McDevitt et al., 2014; Tang et al., 2014; Chan et al., 2016; Gonggalanzi et al., 2016; Cheng et al., 2017; Wu et al., 2018). This is predominantly due to the heterogeneity in study location, design, population, AMS diagnostic tool, and, most importantly, the small number of older adults included in the studies (Wu et al., 2018; Gianfredi et al., 2020). Studies that concluded older age as a protective factor have included more participants who were representative of the age group >55 years (Tang et al., 2014). Contrarily, a study that reported older age as a risk factor for AMS was under-represented by older adults (Gonggalanzi et al., 2016). A recent meta-analysis of 17 studies suggested no association between age and the risk of AMS (Wu et al., 2018), but only four of the 17 papers have included subjects from the older age group—where all 4 papers demonstrated the protective effect of age.

In our study, AMS climbers were younger than non-AMS climbers (31.8 ± 9.6 years vs 36.2 ± 10.9 years, *p* = 0.002); older climbers have significantly lower odds of AMS in the bivariate analysis, and this effect remained significant in the multivariable analysis. The age of our participants ranged from 18 to 75 years. Despite this range, those aged >55 comprised of only 5% of our samples. Therefore, future research should consider quota sampling to ensure an adequate sample size from the older age group for a better estimation of the AMS incidence among the elderly. With a representative sample, the age factor can be analyzed by strata (e.g., young, middle age, and elderly). A better understanding of AMS among different age cohorts will allow risk stratification. This is crucial because more elderlies have opted for high-altitude travel as their retirement past time in recent years (Keyes et al., 2016).

### Other risk factors of AMS in Mount Kinabalu

AMS on day 1 and the lack of guide advice were the risk factors of AMS on day 2 of the Mount Kinabalu climb. The majority of our participants were recreational climbers with low AMS knowledge, and this predisposed them to a higher AMS risk compared with experienced mountaineers (Mairer et al., 2010). We believe that this climbers' composition is not unique to Mount Kinabalu but a common scene in short hikes offered by many famous mountains. Therefore, mountain guides can play a significant role in the provision of pretrek briefing and recognizing the early warning signs of AMS among their clients. Upon symptoms recognition, guides can caution and monitor symptomatic climbers closely to prevent potentially fatal mountain injuries, such as missteps and falls. There is also a need to devise a standardized content for the briefing sessions to ensure that all climbers receive the same advice before their ascent. To adequately care for their clients, guides' knowledge about the risks and proper management of high-altitude exposure is important. However, this study did not explore guides' knowledge regarding AMS, and we suggest future research to look into this.

Alcohol consumption was one of the risk factors of AMS at Mount Kinabalu. Our finding contradicts previous studies that found alcohol consumption not to be a predictor of AMS (Schneider et al., 2002; Mairer et al., 2010; Davis et al., 2016). Some advised against the use of alcohol at a higher altitude as alcohol inhibits acute ventilation adaptation to mild hypoxia and impairs judgment (Roeggla et al., 1995; Klocke et al., 1998; Kim et al., 2016). Mount Kinabalu is popular for tourist hikes, and alcoholic beverages can easily be found at the foothill and mountain lodges. While the association between alcohol consumption and AMS needs further investigation, the national park management should consider discouraging the sale of alcohol to climbers. The management should also work with travel agencies to encourage alcohol abstinence before and during the climb, and create awareness about the effects of alcohol at higher altitudes.

### Factors not associated with AMS in Mount Kinabalu

Ascent rate was not a risk factor for AMS at Mount Kinabalu. The mean ascent time difference between climbers with and without AMS at Mount Kinabalu was diminutive (Δ = 10 minutes, *p* > 0.05). The AMS studies in several short-duration hikes—2 days 1 night or 3 days 2 nights hike at Jade Mountain, Jiaming Lake, and the Eastern Alps—have also reported similar findings (Mairer et al., 2009; Wang et al., 2010; Hsu et al., 2015). Conventionally, fast ascent is a known risk factor for AMS in multiday hikes (Hackett et al., 1976; Schneider et al., 2002), and guidelines have cautioned climbers to make slower ascent (<500 m per day) at an altitude >3000 m to allow for acclimatization (Luks et al., 2010; Imray et al., 2011; Bärtsch and Swenson, 2013). However, we suspect that ascent rate may not be relevant in predicting AMS among individuals climbing Mount Kinabalu or other short treks *per se*, especially when there is a limited option for first overnight altitude before the summit ascent.

In addition, frequent hydration was not protective against AMS at Mount Kinabalu. (Table 4) Our finding corroborates the guideline that suggested that force hydration has no role in AMS prevention and may even result in hyponatremia (Luks et al., 2010). While excessive hydration may be harmful, adequate hydration is important if climbers take acetazolamide for AMS prevention, experience heavy exertion, or climb in a hot climate like Malaysia's. Dehydration is common during strenuous exercise, and the symptoms can mimic those of AMS.

The pulse oximeter is an easy, inexpensive, portable, and noninvasive tool often used at high altitudes to measure pulse rate and the percentage of arterial hemoglobin oxygen saturation of an individual. Despite increased pulse rate and decreased oxygen saturation being the manifestations of AMS, the utility of pulse rate and SpO_2_ value in the diagnosis of AMS remains limited and not encouraged (Chen et al., 2012; Bärtsch and Swenson, 2013). Although laboratory-simulated research (Burtscher et al., 2004) has shown lower SpO_2_ values in AMS-susceptible individuals after being exposed to hypobaric hypoxia conditions, real-world observational research (Connor et al., 2004) has found no difference in the mean pulse rates and SpO_2_ values of AMS and non-AMS climbers. Also, pulse rate and oxygen saturation can be influenced by other factors such as fitness level, ascending time, and pacing technique, thereby further limiting their usefulness in diagnosing AMS. In our study, the mean pulse rate and mean SpO_2_ differed significantly between days 1 and 2, respectively, but the differences lacked clinical significance. Therefore, we discourage the use of pulse rate and SpO_2_ readings to predict AMS at Mount Kinabalu. The diagnosis of AMS should be based on clinical signs and symptoms.

### Other recommendations

Very little is known about AMS at Mount Kinabalu, and much can be done to improve climbers' knowledge and awareness. Bivariate analysis showed that having at least moderate knowledge about AMS reduced the odds of developing AMS. We suggest that official statistics, description of AMS-related injuries, and educational materials be displayed on tourism websites and at resting huts along the trail to remind climbers on the ways to prevent and recognize AMS. Future research should consider evaluating the effectiveness of these interventions in enhancing climbers' knowledge and preventing AMS.

### Limitations and strengths

#### Limitations

First, the questionnaire and measurements were conducted at the Laban Rata basecamp (3272 m) on both days instead of at the last checkpoint of the summit trail (4072 m) on the second day. It could introduce minimal recall bias affecting our estimation of AMS incidence. This limitation was bound by the logistic feasibility at such altitudes. We suggest future research to utilize offline mobile applications and involve mountain guides in data collection, so that the data collection can be completed at a representative altitude. Second, the accuracy of the pulse oximeter could be affected by the environment temperature and poor perfusion of cold fingers among climbers at high altitudes. Third, the measurements of ascent time and water intake were subjective and prone to recall bias. We minimized this bias by allowing climbers to discuss ascent time with their teammates and used water bottles to aid the estimation of water intake. Finally, we were unable to distinguish if climbers took acetazolamide per prophylaxis guidelines. As a result, the insignificant effect of acetazolamide in the bivariate analysis should be interpreted with this limitation in mind.

#### Strengths

We used the revised Lake Louise score 2018 and grading system for the diagnosis of AMS. The revised Lake Louise score, without the sleeping disturbance component, is more accurate at identifying AMS cases (Roach et al., 2018). Next, we reported the values of 95% CI in all of our results, alongside point estimates, for study comparison in the future. Finally, this is the first initiative to report AMS epidemiology data at Mount Kinabalu and to provide local evidence to the national park management to improve AMS awareness.

## Conclusion

Although the incidence of AMS is lower in Mount Kinabalu—22%–24% on both ascent days with a majority of the cases being mild—it should not be treated lightly. Our study showed that younger climbers had higher odds of developing AMS, although more research by age strata is needed to ascertain this association. AMS on day 1, guide advice, and alcohol consumption were significant determinants of AMS on day 2. Ascending time and water intake were not associated with AMS in Mount Kinabalu, and this may change how we advise future climbers. There is a strong call for collaborative efforts from national park management, mountain guides, travel agencies, climbing enthusiasts, and researchers to implement interventions in mitigating the risk of AMS and AMS-related injuries at Mount Kinabalu.

## Ethical Approval

This study was conducted in accordance with the ethical principles outlined in the Declaration of Helsinki and the Malaysian Good Clinical Practice Guideline. The study was registered under the National Medical Research Register (NMRR-18-976-41467) and approved by the Medical Research Ethics Committee, Ministry of Health Malaysia.

## References

[B1] Bärtsch P, and Swenson ER (2013). Acute high-altitude illnesses. N Engl J Med 368:2294–2302 23758234 10.1056/NEJMcp1214870

[B2] Bursac Z, Gauss CH, Williams DK, and Hosmer DW (2008). Purposeful selection of variables in logistic regression. Source Code Biol Med 3:17 19087314 10.1186/1751-0473-3-17 PMC2633005

[B3] Burtscher M, Flatz M, and Faulhaber M (2004). Prediction of susceptibility to acute mountain sickness by SaO_2_ values during short-term exposure to hypoxia. High Alt Med Biol 5:335–340 15453999 10.1089/ham.2004.5.335

[B4] Chan CW, Lin YC, Chiu YH, Weng YM, Li WC, Lin YJ, Wang SH, Hsu TY, Huang KF, and Chiu TF (2016). Incidence and risk factors associated with acute mountain sickness in children trekking on Jade Mountain, Taiwan. J Travel Med 23: tav008 10.1093/jtm/tav008 26782126

[B5] Chen H, Lin W, Wu J, Wang S, and Chiu T (2012). Change in oxygen saturation does not predict acute mountain sickness on Jade Mountain. Wilderness Environ Med 23:122–127 22656657 10.1016/j.wem.2012.03.014

[B6] Cheng FY, Jeng MJ, Lin YC, Wang SH, Wu SH, Li WC, Huang KF, and Chiu TF (2017). Incidence and severity of acute mountain sickness and associated symptoms in children trekking on Xue Mountain, Taiwan. PLoS One 12:e0183207 28832689 10.1371/journal.pone.0183207 PMC5568320

[B7] Connor TO, Dubowitz G, and Bickler PE (2004). Pulse oximetry in the diagnosis of acute mountain sickness. High Alt Med Biol 5:341–348 15454000 10.1089/ham.2004.5.341

[B8] Davis C, Reno E, Maa E, and Roach R (2016). History of migraine predicts headache at high altitude. High Alt Med Biol 17:300–304 27788038 10.1089/ham.2016.0043

[B9] Gianfredi V, Albano L, Basnyat B, and Ferrara P (2020). Does age have an impact on acute mountain sickness? A systematic review. J Travel Med: taz104. [Epub ahead of print] DOI: 10.1093/jtm/taz104 31897482

[B10] Gonggalanzi Labasangzhu, Nafstad P, Stigum H, Wu T, Haldorsen OD, Ommundsen K, and Bjertness E (2016). Acute mountain sickness among tourists visiting the high-altitude city of Lhasa at 3658 m above sea level: A cross-sectional study. Arch Public Health 74:23 27252854 10.1186/s13690-016-0134-z PMC4888367

[B11] Hackett PH, Rennie D, and Levine HD (1976). The incidence, importance, and prophylaxis of acute mountain sickness. Lancet 308:1149–1155 10.1016/s0140-6736(76)91677-9 62991

[B12] Horiuchi M, Endo J, Akatsuka S, Uno T, and Jones TE (2016). Prevalence of acute mountain sickness on Mount Fuji: A pilot study. J Travel Med 23: taw024 10.1093/jtm/taw024 27147731

[B13] Hsu TY, Weng YM, Chiu YH, Li WC, Chen PY, Wang SH, Huang KF, Kao WF, Chiu TF, and Chen JC (2015). Rate of ascent and acute mountain sickness at high altitude. Clin J Sport Med 25:95–104 24751723 10.1097/JSM.0000000000000098

[B14] Imray C, Booth A, Wright A, and Bradwell A (2011). Acute altitude illnesses. BMJ 343: d4943 10.1136/bmj.d4943 21844157

[B15] Kao WF, Kuo CC, Hsu TF, Chang H, Sung YY, Yen DHT, Wu JK, and Lee CH (2002). Acute mountain sickness in Jade Mountain climbers of Taiwan. Aviat Space Environ Med 73:359–362 11952056

[B16] Keyes LE, Mather L, Duke C, Regmi N, Phelan B, Pant S, Starling J, McElwee M, Cole D, McConnell T, Paudel P, Sallade TD, Sheets A, Twillman D, Young DS, and Basnyat B (2016). Older age, chronic medical conditions and polypharmacy in Himalayan trekkers in Nepal: An epidemiologic survey and case series. J Travel Med 23: taw052 10.1093/jtm/taw052 27503853

[B17] Kim SB, Kim JS, Kim SJ, Cho SH, and Suh DC (2016). Altitude stress during participation of medical congress. Neurointervention 11:73 27621942 10.5469/neuroint.2016.11.2.73 PMC5018551

[B18] Klocke DL, Decker WW, and Stepanek J (1998). Altitude-related illnesses. Mayo Clin Proc 73:988–993 9787751 10.4065/73.10.988

[B19] Luks AM, McIntosh SE, Grissom CK, Auerbach PS, Rodway GW, Schoene RB, Zafren K, and Hackett PH; Wilderness Medical Society (2010). Wilderness medical society consensus guidelines for the prevention and treatment of acute altitude illness. Wilderness Environ Med 21:146–155 20591379 10.1016/j.wem.2010.03.002

[B20] Maeder MB, Brugger H, Pun M, Strapazzon G, Cappello TD, Maggiorini M, Hackett P, Bärtsch P, Swenson ER, and Zafren K (2018). The STAR data reporting guidelines for clinical high altitude research. High Alt Med Biol 19:7–14 29596018 10.1089/ham.2017.0160 PMC5905862

[B21] Mairer K, Wille M, Bucher T, and Burtscher M (2009). Prevalence of acute mountain sickness in the eastern Alps. High Alt Med Biol 10:239–245 19775213 10.1089/ham.2008.1091

[B22] Mairer K, Wille M, and Burtscher M (2010). The prevalence of and risk factors for acute mountain sickness in the Eastern and Western Alps. High Alt Med Biol 11:343–348 21190503 10.1089/ham.2010.1039

[B23] McDevitt M, McIntosh SE, Rodway G, Peelay J, Adams DL, and Kayser B (2014). Risk determinants of acute mountain sickness in trekkers in the Nepali Himalaya: A 24-year follow-up. Wilderness Environ Med 25:152–159 24864065 10.1016/j.wem.2013.12.027

[B24] Ranganathan P, Pramesh C, and Aggarwal R (2017). Common pitfalls in statistical analysis: Logistic regression. Perspect Clin Res 8:148–151 28828311 10.4103/picr.PICR_87_17 PMC5543767

[B25] Roach RC, Bartsch P, Oelz O, and Hackett P (1993). The Lake Louise acute mountain sickness scoring system. Hypoxia Mol Med 272–274

[B26] Roach RC, Hackett PH, Oelz O, Bärtsch P, Luks AM, Macinnis MJ, and Baillie JK; Lake Louise AMS Score Consensus Committee (2018). The 2018 Lake Louise acute mountain sickness score. High Alt Med Biol 19:2017–2019 10.1089/ham.2017.0164 PMC6191821 29583031

[B27] Roeggla G, Roeggla H, Roeggla M, Binder M, and Laggner AN (1995). Effect of alcohol on acute ventilatory adaptation to mild hypoxia at moderate altitude. Ann Intern Med 122:925–927 7755228 10.7326/0003-4819-122-12-199506150-00006

[B28] Schneider M, Bernasch D, Weymann J, Holle R, and Bartsch P (2002). Acute mountain sickness: Influence of susceptibility, pre-exposure and ascent rate. Med Sci Sport Exerc 34:1886–1891 10.1097/00005768-200212000-00005 12471292

[B29] Tang XG, Zhang JH, Qin J, Gao X Bin, Li QN, Yu J, Ding XH, and Huang L (2014). Age as a risk factor for acute mountain sickness upon rapid ascent to 3,700 m among young adult chinese men. Clin Interv Aging 9:1287–1294 25120358 10.2147/CIA.S67052 PMC4128797

[B30] Waeber B, Kayser B, Dumont L, Lysakowski C, Trame MR, and Elia N (2015). Impact of study design on reported incidences of acute mountain sickness: A systematic review. High Alt Med Biol 16:204–215 26230550 10.1089/ham.2015.0022

[B31] Wang S, Chen Y, Kao W, Lin Y, Chen J, Chiu T, Hsu TY, Chen HC, and Liu SW (2010). Epidemiology of acute mountain sickness on Jade Mountain, Taiwan: An annual prospective observational study. High Alt Med Biol 11:43–49 20367488 10.1089/ham.2009.1063

[B32] Wu Y, Zhang C, Chen Y, and Luo Y (2018). Association between acute mountain sickness (AMS) and age: A meta-analysis. Mil Med Res 5:1–8 29747689 10.1186/s40779-018-0161-x PMC5946480

